# Repression of PLA2R1 by c-MYC and HIF-2alpha promotes cancer growth

**DOI:** 10.18632/oncotarget.1681

**Published:** 2014-01-16

**Authors:** David Vindrieux, Guillaume Devailly, Arnaud Augert, Benjamin Le Calvé, Mylène Ferrand, Pascal Pigny, Léa Payen, Gérard Lambeau, Michael Perrais, Sébastien Aubert, Hélène Simonnet, Robert Dante, David Bernard

**Affiliations:** ^1^ Inserm U1052, Centre de Recherche en Cancérologie de Lyon, Lyon, France; ^2^ CNRS UMR5286, Lyon France; ^3^ Centre Léon Bérard, Lyon, France; ^4^ Université de Lyon, Lyon, France; ^5^ INSERM U837, Jean-Pierre Aubert Research Center, Lille, France; ^6^ Institut de Biochimie et Biologie Moléculaire Centre de Biologie Pathologie CHRU Lille, Lille, France; ^7^ Institut de Pharmacologie Moléculaire et Cellulaire, UMR6097, CNRS and Université de Nice-Sophia Antipolis, Valbonne, France; ^8^ Institut de Pathologie, CHRU, Faculté de Médecine, Université de Lille, Lille, France

**Keywords:** VHL, MYC, HIF, PLA2R1, tumor suppressor

## Abstract

Loss of secreted phospholipase A2 receptor (PLA2R1) has recently been found to render human primary cells more resistant to senescence whereas increased PLA2R1 expression is able to induce cell cycle arrest, cancer cell death or blockage of cancer cell transformation in vitro, suggesting that PLA2R1 displays tumor suppressive activities. Here we report that PLA2R1 expression strongly decreases in samples of human renal cell carcinoma (RCC). Knockdown of PLA2R1 increases renal cancer cell tumorigenicity supporting a role of PLA2R1 loss to promote in vivo RCC growth. Most RCC result from Von Hippel-Lindau (VHL) tumor suppressor loss-of-function and subsequent gain-of-function of the oncogenic HIF-2alpha/c-MYC pathway. Here, by genetically manipulating VHL, HIF-2alpha and c-MYC, we demonstrate that loss of VHL, stabilization of HIF-2alpha and subsequent increased c-MYC activity, binding and transcriptional repression, through induction of PLA2R1 DNA methylation closed to PLA2R1 transcriptional start site, results in decreased PLA2R1 transcription. Our results describe for the first time an oncogenic pathway leading to PLA2R1 transcriptional repression and the importance of this repression for tumor growth.

## INTRODUCTION

The secreted phospholiapse A2 receptor (PLA2R1) belongs to the mannose receptor family. It binds several secreted phospholipases A2 (sPLA2s) and various collagens and carbohydrates [1-5]. We have previously discovered that in normal human cells, loss of the phospholipase A2 receptor (PLA2R1) delays senescence or favors senescence escape, depending on the stress [6, 7], senescence being a tumor suppressive pathway [8-13]. In addition, PLA2R1 knockout mice are more prone to form tumors induced by RAS [7]. These tumor suppressive effects of PLA2R1 are at least in part mediated by increased JAK2 activity and increased ROS production [6, 7, 14]. Together these results strongly support a tumor-suppressive activity of PLA2R1. Nevertheless, the mechanisms regulating PLA2R1 expression are currently unknown.

Analysis in the Oncomine database, a cancer microarray database allowing to perform differential expression analyses comparing most major types of cancer with respective normal tissues, shows that numerous cancers display an unusually low PLA2R1 mRNA level [7]. Amongst these cancers, kidney cancers are the one displaying the strongest decrease. In addition, PLA2R1 has recently been described as an important protein in kidney physiopathology [15], we have then decided to investigate whether it might play a tumor-suppressive role in kidney cancer. The most frequent and aggressive renal cancer is conventional renal cancer or clear cell Renal Cell Carcinoma (RCC). These highly vascularized tumors, either sporadic or familial, are strongly associated with biallelic inactivation of the Von Hippel-Lindau gene (*VHL*), by point mutation, loss of heterozygosity, and/or promoter methylation [16]. The VHL tumor suppressor is an E3-ubiquitin ligase recognizing regulatory proteins such as the alpha subunits of the Hypoxia Inducible Factors (HIFs), the interaction being followed, generally, by ubiquitination and proteasomal degradation of the partner. Loss of VHL in RCC thus results in increased HIFα stability. This stabilization of HIFα proteins, particularly HIF2α, is thought to be a driving force of RCC initiation and progression [17, 18].

We have thus investigated the possibility that PLA2R1 might play a role in regulating kidney cell transformation and RCC growth and have examined how its expression might be related to the VHL pathway.

## RESULTS

### PLA2R1 expression is lost in clear cell renal cell carcinoma

**Figure 1:**
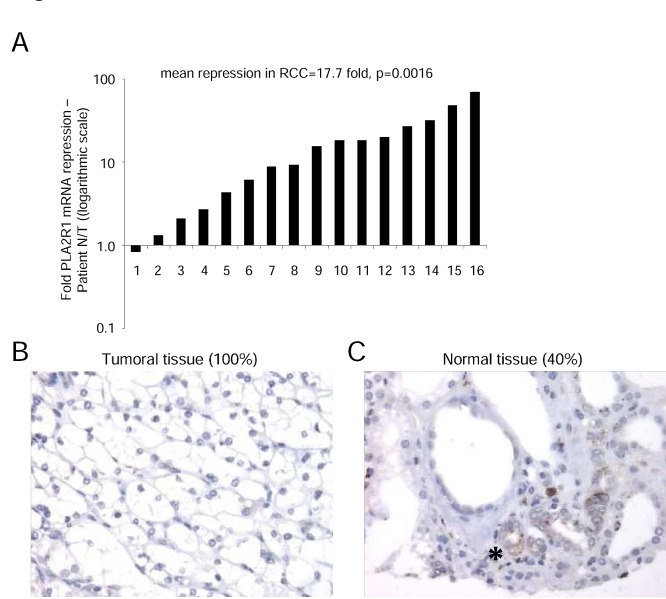
PLA2R1 expression decreases in clear cell renal cell carcinoma (A) RNAs were extracted from both normal and cancer tissues from the same patients (n=16) and PLA2R1 transcript levels were assayed by qRT-PCR and normalized against PPIA. (B) Photograph representative of 100% of the analyzed samples (n=27), PLA2R1 immunostaining of RCC. (C) Photograph representative of 40% of the analyzed samples (n=27), PLA2R1 immunostaining of normal renal tubules. The asterisk shows the PLA2R1 staining in some normal renal tubules.

To examine whether PLA2R1 might exert tumor-suppressive activity in RCC, we first analyzed transcript-level PLA2R1 expression in normal and RCC tissue samples. Strikingly, the PLA2R1 mRNA level was markedly lower in RCC tissue samples than in their normal counterparts (Figure 1A). This transcript-level down regulation led us to look at the protein level. Using a tissue microarray comprising twenty-seven pT3 RCC tumor tissue samples as described in [21], we found PLA2R1 to be undetectable in all the RCC tumor tissues (Figure 1B). In contrast, the protein was detected in the normal tissue in 40% of the examined samples, in the cells from which RCC originates: cortical tubular epithelial renal cells (Figure 1C) [25].

### A PLA2R1 decrease accelerates RCC growth in mice

**Figure 2:**
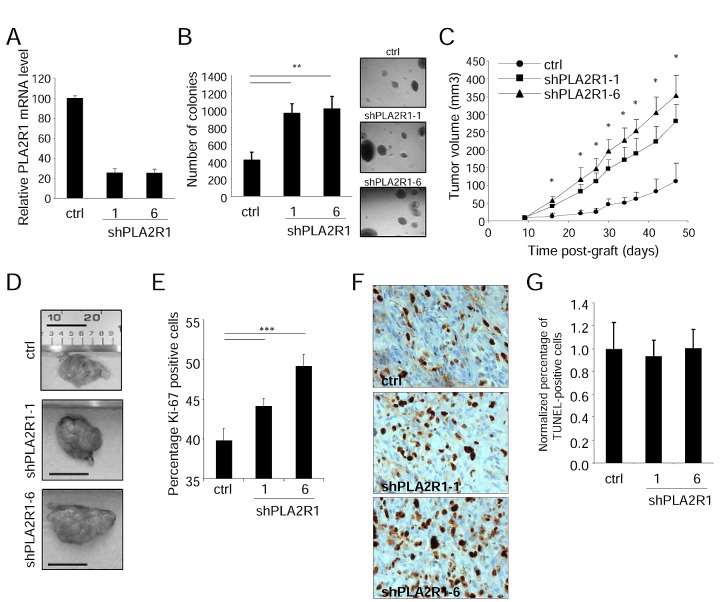
PLA2R1 knockdown favors RCC-derived cell tumorigenicity The ACHN cell line was infected with a control or PLA2R1-targeting shRNA and infected cells were puromycin selected. (A) RNAs were prepared and reverse-transcribed. Quantitative PCR was performed to measure the relative PLA2R1 mRNA level, normalized with respect to the ACTB level, in each indicated condition. (B) Twenty-five thousand ACHN cancer cells were seeded into agar and allowed to grow for 2 weeks. After 2 weeks, colonies were counted for each indicated condition and representative photographs are displayed (mean±SD, 3 independent experiments). (C) Five million ACHN cells infected with a control or PLAR1-targeting shRNA vector were grafted into the right flanks of 7-week-old nude mice. Tumor growth was monitored with a caliper. Tumor volume was calculated as V= LxlxH/2. Statistical analysis was performed with a T-test and the results are shown in Supplementary Figure 1. (D) Photographs show for each condition a representative picture of a tumor at necropsy. Scale bar represents 1 cm. (E-G), Control and PLA2R1-knockdown ACHN-derived tumors were collected, fixed, and embedded. Tumor tissue sections were used to analyze proliferation and cell death for each experimental condition. (E) Immunohistochemical detection of the Ki-67 proliferation marker. Nuclei were counterstained with hematoxylin. Ki-67-positive cells were counted in at least four independent fields per tumor in at least three independent tumors. (F) Representative photographs are shown. (G) TUNEL assays were performed to label dying cells. After Hoechst staining of the nuclei, the percentage of TUNEL-positive cells was calculated for each condition and normalized with respect to the control value (at least 3 independent tumors and at least 4 independent fields/tumor were analyzed).

As PLA2R1 levels strongly decreased in RCC, we next examined whether PLA2R1 down regulation might influence RCC tumor growth. To this end we generated stable PLA2R1-knockdown ACHN cells, using two different PLA2R1-targeting shRNAs (Figure 2A). PLA2R1-knockdown ACHN cells displayed increased transformation ability, as demonstrated by their capacity to form more and bigger colonies than control ACHN cells in soft agar (Figure 2B). Next, control or PLA2R1 knockdown ACHN cells were injected subcutaneously into nude mice and tumor growth was monitored for 47 days after grafting. PLA2R1 knockdown was found to increase tumor growth significantly, the average tumor volume reaching about three times that of control tumors (Figure 2C-D and Supplementary Figure S1).

We next investigated the mechanism of this tumor growth acceleration by analyzing proliferation and cell death in the xenograft tumors. Ki-67 staining revealed a significant increase in proliferation in response to PLA2R1 depletion (Figure 2E-F), whereas TUNEL assays revealed no impact of PLA2R1 depletion on cell death (Figure 2G).

### Constitutive PLA2R1 expression inhibits RCC growth in mice

**Figure 3:**
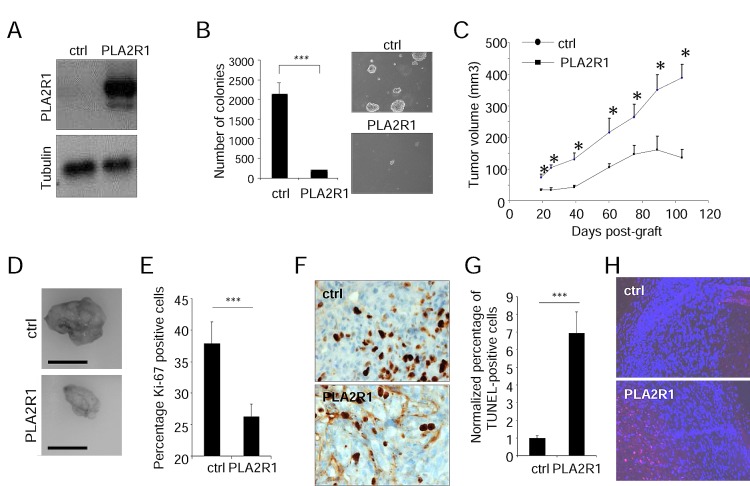
PLA2R1 expression inhibits transformation and decreases tumor growth ACHN cells were infected with a control or PLA2R1-encoding vector. Infected cells were next puromycin selected. (A) Ectopic expression of PLA2R1 was analyzed by western blotting. Tubulin was used as a loading control. (B) Twenty-five thousand ACHN cells were seeded in agar and allowed to grow. After 2 weeks, colonies were counted for each indicated condition and representative photographs are displayed (mean±SD, 3 independent experiments). Histograms show an experiment representative of 3 independent experiments. Statistics were performed with the T-test. *** represents a p value< 0.005. (C) Five million ACHN cells were grafted into the right flanks of 7-week-old nude mice. Tumor growth was monitored with a caliper. Tumor volume was calculated as V= LxlxH/2. Statistical analysis was performed with a T-test and the results are shown in Supplementary Figure 3. (D) Photographs show a picture of tumors at necropsy for each condition. Scale bar represents 1cm. (E-H) Control and PLA2R1-expressing ACHN-derived tumors were collected, fixed, and embedded. Tissue sections from tumors were then used to analyze proliferation and cell death. (E-F) Immunohistochemistry against Ki-67 proliferation marker was performed. Nuclei were counterstained by an hematoxylin solution. (E) Histogram representing the percentage of Ki-67 positive cells was displayed. Positive cells were counted on at least 4 independent fields per tumor and at least 3 independent tumors. (F) Representative photographs are shown. (G-H) TUNEL assays were performed to label dying cells. After nuclear staining with Hoechst dye, the percentage of TUNEL positive cells was calculated (at least 3 independent tumors and at least 4 independent fields/tumor analyzed). (G) TUNEL-positive cell percentages are presented relatively to control cells. (H) Images are shown.

The above observation that decreasing PLA2R1 improves growth of RCC tumors led us to examine the effect of constitutively expressing PLA2R1 in RCC cell lines (ACHN and 786-O). We first infected these cells with a control (ctrl) or a PLA2R1 encoding retroviral vector and checked for constitutive PLA2R1 expression (Figure 3A and Supplementary Figure S2A). Constitutive PLA2R1 expression was found to strongly inhibit the ability of ACHN and 786-O cells to form clones in soft agar (Figure 3B and Supplementary Figure S2B). 786-O-derived tumors grew rapidly in mice, but this tumor growth was slowed down when PLA2R1 was constitutively expressed (Supplementary Figure S2C-E). Importantly, the slow growing ACHN derived tumors responded strongly to constitutive PLA2R1 expression: after reaching a mean size of 161 mm3, they were found to regress (Figure 3C-D and Supplementary Figure S3). We conclude that constitutive PLA2R1 expression delays or blocks RCC xenograft growth, depending on the implanted cells.

In 786-O and ACHN derived tumors, constitutive expression of PLA2R1 was found to affect tumor growth both by decreasing cell proliferation (as estimated by Ki-67 staining, Figure 3E-F and Supplementary Figure S4A-B) and increasing cell death (as estimated in TUNEL assays, Figure 3G-H and Supplementary Figure S4C-D). Together, these data provide the first evidence that increasing the PLA2R1 level causes tumor-suppressive effects in RCC.

### VHL loss of function decreases PLA2R1 levels in RCC

**Figure 4:**
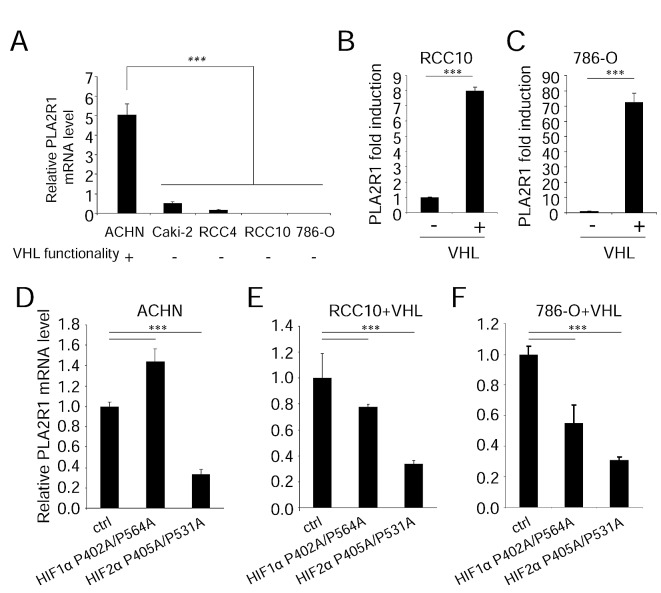
VHL regulates PLA2R1 expression in RCC-derived cells (A) PLA2R1 transcript levels were assayed by qRT-PCR and normalized against ACTB in five RCC cell lines: ACHN, Caki-2, RCC4, RCC10, and 786-O. VHL function is indicated for each cell line. (B) PLA2R1 transcript levels were assayed by qRT-PCR and normalized against ACTB in 786-O and (C) RCC10 control or constitutively expressing VHL. Histograms show an experiment representative of three independent experiments. (D-F) Cells were infected with a control retroviral vector or one encoding a VHL-resistant HIF1α mutant or a VHL-resistant HIF2α mutant. Infected cells were puromycin-selected. RNAs were prepared and reverse transcribed. Quantitative PCR was performed against PLA2R1 and normalized with respect to the ACTB mRNA level in (D) ACHN cells, (E) in RCC10+VHL cells and (F) in 786-O+VHL cells as indicated.

Having observed that PLA2R1 is lost in human RCC samples and that PLA2R1 deregulation strongly impacts RCC tumor growth, we next investigated the mechanism regulating PLA2R1 expression in RCC. The development of most RCCs involves inactivation of the VHL tumor suppressor gene by one or more of the following mechanisms: genetic mutation in the coding region, loss of heterozygosity, promoter hypermethylation [16]. As the twenty-seven RCC samples examined in the TMA showed no detectable PLA2R1 protein, we next investigated whether PLA2R1 might be a downstream target of VHL. First we measured PLA2R1 mRNA levels in five characterized RCC cell lines. ACHN cancer cells are known to express a functional VHL, whereas Caki-2, 786-O, RCC4, and RCC10 cells express a mutated nonfunctional VHL protein. Interestingly, a higher PLA2R1 mRNA level was found in VHL functional ACHN cells than in any of the VHL nonfunctional cancer cell lines (Figure 4A).

Functional confirmation that PLA2R1 is positively regulated by VHL was obtained by engineering VHL negative RCC10 and 786-O cells to express the VHL gene constitutively (the resulting cells are respectively named RCC10+VHL and 786-O+VHL cells): both engineered RCC cell lines showed an increased PLA2R1 transcript level (Figure 4B-C).

VHL exerts its tumor-suppressive activity by inducing degradation of HIFα proteins, but it might also exert HIF-independent activities [17, 18, 26]. To determine whether HIFα protein degradation is involved in VHL-induced PLA2R1 transcription, we examined PLA2R1 transcript levels in ACHN (Figure 4D), RCC10+VHL (Figure 4E), and 786-O+VHL cells (Figure 4F) constitutively synthesizing a non-degradable form of either HIF1α or HIF2α [20]. Constitutive expression of HIF2α strongly reduced PLA2R1 mRNA level in all three cases. Forced HIF1α showed less marked effects (Figure 4D-F).

Together these data demonstrate that PLA2R1 expression is regulated by VHL, and that VHL loss of function in RCC results in PLA2R1 loss of expression, mainly through an HIF2α dependent mechanism.

### An HIF2α-MYC repressive pathway, downstream of VHL loss of function, mediates PLA2R1 repression in RCC

**Figure 5:**
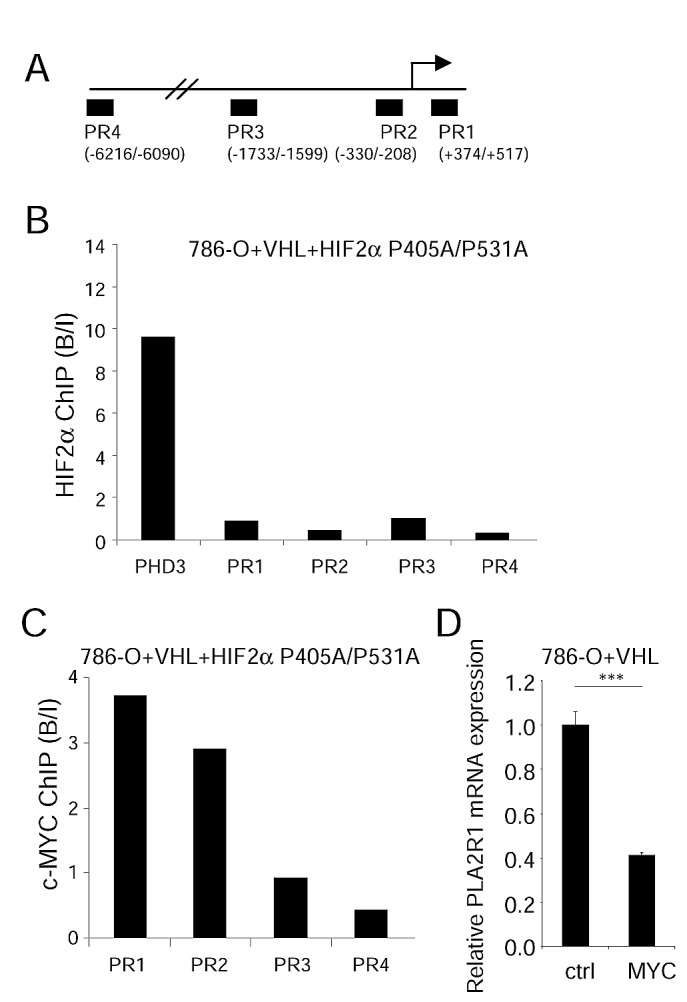
An HIF2α-MYC pathway represses PLA2R1 expression (A) Schematic representation of the PLA2R1 genomic regions tested for HIF2α binding. (B) Binding of HIF2α on PLA2R1 genomic regions was assayed by chromatin immunoprecipitation. ChIP was performed on 786-O+VHL+HIF2α cells and PCR performed on the input and bound fractions. PHD3 was used as positive control for HIF2α binding and 4 genomic regions of PLA2R1 were assayed. (C) Binding of c-MYC on PLA2R1 genomic regions was assayed by chromatin immunoprecipitation. ChIP was performed on 786-O+VHL+HIF2α cells and PCR performed on the input and bound fractions. Four genomic regions of PLA2R1 (PR1 to PR4) were assayed. (D) 786-O+VHL cells were infected with a control or a c-MYC encoding retroviral vector and puromycin selected. Five days later, RNA were prepared and retro transcribed. Quantitative PCR was performed against PLA2R1 and normalized with respect to the ACTB mRNA level.

Even if HIF2α is generally described as a transcriptional activator, it has also been reported to repress transcription [27]. Chromatin immunoprecipitation (ChIP) experiments performed on 4 independent PLA2R1 genomic regions (Figure 5A) revealed no direct binding of HIF2α, whereas and as expected it was found to bind PHD3 [23], a direct HIF2α target (Figure 5B).

HIF2α exerts its oncogenic activity by increasing c-MYC transcriptional activities, the activating as well as the repressing ones [28, 29]. To check whether c-MYC might be a downstream effecter of HIF2α to repress PLA2R1 expression, we immunoprecipitated c-MYC DNA-bound regions in HIF2α-overexpressing cells, cells in which PLA2R1 expression is repressed (Figure 4F). Interestingly, c-MYC was bound to PLA2R1 genomic regions closed to the transcriptional start site (PR1 and PR2) and not to distal PLA2R1 genomic regions (PR3 and PR4) (Figure 5C). Further supporting a repressive role of c-MYC on PLA2R1 expression, its constitutive expression was found to inhibit PLA2R1 expression in 786-O+VHL cells, cells in which PLA2R1 is induced (Figure 5D). These data demonstrate a pathway involving loss of VHL, increased HIF2α and c-MYC activity leading to PLA2R1 repression in RCC.

### The VHL-HIF-MYC pathway regulate PLA2R1 expression through DNA methylation

C-MYC represses transcription by various mechanisms including DNA methylation [30, 31]. Interestingly, PLA2R1 has recently been shown to be repressed by DNA methylation in leukemic cells [32] pointed out a potential mechanism by which the VHL-HIF2α-MYC pathway might regulate PLA2R1 expression in RCC.

**Figure 6:**
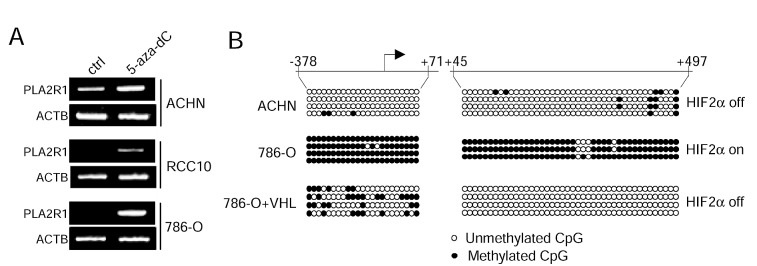
The VHL-HIF2α-MYC regulates PLA2R1 expression through its DNA methylation (A) Indicated RCC-derived cell lines were treated with or without 5-aza-dC at 5μM during 24 hrs. RNA were prepared, retro-transcribed and PCR performed against PLA2R1 or ACTB as loading control using the primers described in [7]. (B) Genomic DNA of the indicated cells were prepared and subjected to sodium bisulphate modification. PCR was performed to amplify the indicated regions. The amplified regions were subcloned and sublconed fragments were sequenced to determine whether or not the CpG were methylated.

To know whether the expression of PLA2R1 might be regulated by DNA methylation, we treated VHL-positive (ACHN) and -negative (RCC10 and 786-O) derived RCC cell lines with the 5-azadeoxycytidine (5-aza-dC) DNA methyl transferase (DNMT) inhibitor. Both VHL-negative cell lines displayed a strong PLA2R1 induction upon DNMT inhibition by contrast to the VHL-positive cell line (Fig. 6A). This suggests that the repressive VHL-HIF2α-MYC pathway regulates PLA2R1 expression through DNA methylation.

We next carried out bisulphite genomic sequencing to compare PLA2R1 methylation in ACHN cells (VHL-positive, PLA2R1-positive), 786-O cells (VHL-negative, PLA2R1-negative) and 786-O+VHL cells (PLA2R1 positive). CpG methylation were examined in a PLA2R1 region spending from -378 to +497, a region overlapping the c-MYC binding regions as seen in the figure 5C. CpG island hypermethylation was observed in PLA2R1-negative 786-O cells. By contrast, this island was not methylated after forced VHL expression in these cells or in VHL-positive ACHN cells further suggesting that this repressive pathway is acting by controlling DNA methylation on PLA2R1 promoter.

## DISCUSSION

Clear cell renal cell carcinoma represents 80% of all renal cancers and is the most aggressive and deadly one. We show here that PLA2R1 is lost in RCC and that this loss might be an important event increasing RCC growth as its decrease accelerates tumor growth in nude mice. We have also examined the effect of using cells constitutively expressing PLA2R1 in nude-mouse xenograft RCC models, using two different RCC-derived cell lines (ACHN or 786-O) to generate the tumors. In both cases the implanted cells show a reduced tumor-promoting potential, due both to induce cell death and slower proliferation. Most interestingly, the slow-growing ACHN-derived tumors, after some initial tumor growth, are regressing. This suggests that restoring this pathway might be an effective way to trigger RCC regression.

A major step in the initiation of the RCC is the *VHL* tumor-suppressor gene inactivation. PLA2R1 expression correlates with loss of VHL tumor-suppressor function, and restoring VHL expression is sufficient to restore PLA2R1 expression in RCC-derived cell lines. Our observation support the view that this effect of VHL depends mainly on its ability to degrade its HIF2α target and not HIF1α. HIF2α and HIF1α are both down regulated by VHL. Although it was initially thought that the two proteins share common pro-tumor functions, recent data suggest some specificity for each protein, in terms of transcriptional targets and interacting proteins [33]. Importantly HIF2α, unlike HIF1α, is now viewed as a key oncogenic actor in RCC, particularly through its ability to increase c-MYC activity [28, 29].

HIF2α is unable to bind PLA2R1 genomic regions suggesting that HIF2α indirectly represses PLA2R1. Transcriptional repression by c-MYC of genes blocking tumor growth such as p21 and p27 CKIs is part of its pro tumoral program [28]. c-MYC, which is known to mediate HIF2α oncogenic effect, is thus a good candidate to mediate HIF2α-induced PLA2R1 repression. Confirming this hypothesis, c-MYC binds PLA2R1 genomic regions closed to PLA2R1 transcription start site and its forced expression represses PLA2R1 expression. C-MYC is known to repress transcription by recruiting various repressive complexes including the DNA methyl transferases (DNMT), the enzymes that methylate the CpG [30, 31] and PLA2R1 has recently been found to be methylated,[32] suggesting that c-MYC might exert its repressive activity through induction of PLA2R1 DNA methylation. Indeed, inhibiting the DNMT in cells expressing low levels of PLA2R1 is sufficient to restore PLA2R1 expression. In addition, PLA2R1 promoter methylation is observed when VHL is not functional; then when the HIF2α-MYC pathway exerts its repressive activity. By contrast, PLA2R1 CpG island methylation is lost when VHL is functional; then when the HIF2α-MYC pathway is off and unable to exert its repressive activity on PLA2R1.

In conclusion, our study report novel findings showing that PLA2R1 is repressed in RCC by the loss of VHL tumor suppressor and the activation of the oncogenic HIF2α-c-MYC pathway and that repression favors RCC tumorigenecity.

## MATERIALS AND METHODS

### Cell Culture

The human kidney cancer cell lines ACHN, Caki-2, RCC4, RCC10, and 786-O were obtained from ATCC and virus-producing GP293 cells from Clontech. VHL-expressing RCC4, RCC10, and 786-O cells have been described in [19]. Cell lines were cultured in DMEM (Invitrogen) containing Glutamax and supplemented with 10% FBS (Lonza) and 1% penicillin/streptomycin (Invitrogen). Upon receipt, cells were thawed, amplified and aliquots frozen. Experiments were performed from these aliquots within a 4 months period without further authentication of the cell lines.

### Vectors

Human wild-type membrane-bound PLA2R1 (GenBank NM 007366) was generated by PCR from the PLA2R1-encoding pSupF vector and ligated into the pGEMTeasy vector (Promega), fully sequenced, and subsequently subcloned in the pLPCX retroviral vector (Clontech) between XhoI/NotI restriction sites. PLA2R1-shRNA-encoding retroviral vectors have been described in [6]. The following vectors were supplied by Addgene: pBabe-VHL [19] (# 19234), pBabe-HIF-1α P402A/P564A (#19005), pBabe-HIF2αP405A/P531A (#19006) [20].

### Immunohistochemistry

The RCC study was performed on 4-μm-thick sections from a tissue microarray comprising 27 formalin- and paraffin-embedded RCC tumor tissue samples, each with the surrounding morphologically benign-appearing renal parenchyma.[21] Heat antigen retrieval was done in citrate buffer pH 6 in a pressure cooker. Endogenous peroxidase was blocked by addition of H2O2. Incubation with PLA2R1 antibody was carried out overnight at 4°C. In the negative control, the primary antibody was omitted [15]. In the xenograft experiments, fresh tumors were fixed for 48 h in 10% neutral buffered formalin (Sigma). Paraffin-embedded sections were treated with unmasking solution (VectorLABS) and exposed to H2O2 to block endogenous peroxidase activity. For Ki-67 staining, tumor sections were incubated with an antibody directed against Ki-67 antigen (Dako), a biotinylated secondary antibody (Dako), and streptavidin-HRP solution (Dako). Nuclei were counterstained with hematoxylin solution (Sigma) and analyzed by light microscopy. For TUNEL assays, tumor sections were treated with 4% PFA and 0.2% PBS-Triton and then incubated with TdT enzyme and biotinylated dUTP (Roche) according to the manufacturer's instructions. Biotinylated DNA was detected with Cy-3-coupled streptavidin (Interchim). Nuclei were stained with Hoechst dye (Sigma). Samples were analyzed by fluorescence microscopy.

### Soft-agar and xenograft experiments

To measure anchorage-independent growth, cells were detached with trypsin and suspended in growth medium. Base agar was prepared with 0.75% low-melting agarose (Lonza) in growth medium. The top agar contained the cells suspended in 0.45% low-melting agarose (1.25-2.5x10^4^ cells/well in 6-well plates, for 786-O and ACHN respectively). Plates were incubated for 1 (786-0) or 2 weeks (ACHN) and colonies were counted under a bright light microscope.

For the xenograft experiments, 5x10^6^ control or modified cells in 200 μl PBS/Matrigel (BD Biosciences) (3/1, v/v) were injected subcutaneously into the right flanks of 6-week-old female athymic nude mice (nu/nu). Tumor size was measured with an electronic caliper, weekly or at sacrifice. Tumor volume (V) was calculated using the following formula: V=axbxc/2, where “a” is the longest diameter, “b” is the shortest one, and c the depth. Numbers of mice with size details and statistics are indicated in Supplementary Figures. The mice were housed and bred in the specific pathogen-free animal facility AniCan at the CRCL, Lyon, France. The experiments were performed in accordance with the animal care guidelines of the European Union and with French legislation and were approved by the local Animal Ethics Evaluation Committee (CECCAPP).

### Transfection and infection

To transfect GP293 cells, PEI reagent was used according to the manufacturer's recommendations (Euromedex). Two days after transfection, the viral supernatant mixed with fresh medium (1/2) and polybrene (final concentration: 8ug/mL) was used to infect target cells. The cells were infected for 12-24 h depending on the cell type. Importantly, the infection protocols were designed so that practically all the cells were infected, as judged by the results of infection with a GFP control. One day post-infection, cells of each cell line used were selected with the appropriate antibiotic at the final concentration of 500 ng/ml.

### Immunoblots

Cell lysates were prepared in Laemmli buffer (50mM Tris·HCl, pH 7.4, 250mM NaCl, 0.2% Triton X-100, 5mM EDTA) supplemented with protease and phosphatase inhibitors (Roche). Lysates were clarified by centrifugation at 14,000 rpm for 30 min at 4°C. Protein concentrations were measured by means of the Bradford protein assay (Biorad #500-0006). Cell extracts were resolved by SDS-PAGE under non-reducing conditions for PLA2R1 detection or reducing conditions for detection of other proteins and transferred onto nitrocellulose membranes. For detection, the following primary antibodies were used: anti-PLA2R1 [15], anti-tubulin (T6199, Sigma). After overnight incubation at 4°C with primary antibodies, the blots were washed with PBS containing 0.05% Tween, incubated with a peroxidase-coupled secondary antibody, and washed again in PBS containing 0.05% Tween. Antigen-antibody complexes were detected by ECL (Amersham).

### RNA extraction, reverse transcription, and PCR

Normal and tumoral human kidney tissues were provided by the biobank of the CHU of Lille. Phenol-chloroform total RNA extraction was performed with TriReagent (Sigma-Aldrich, Saint Louis, MO, USA). PhaseLockGel tubes (Eppendorf, Hamburg, Germany) were used for phase separation. cDNA was synthesized from 3 μg total RNA with the First-Strand cDNA Synthesis Kit (GE Healthcare, Chalfont St Giles, UK). The RT reaction mixture was diluted 1/60 and used as cDNA template for qPCR analysis. TaqMan quantitative PCR analysis was carried out on a LightCycler 2.0 System (Roche Applied Science). PCR mixtures contained LightCycler TaqMan mix, 200 nM primers, and 1.67 μl cDNA template in a 6.67-μl reaction volume. The PPIA or ACTB housekeeping gene, as indicated, was used in kidney samples for normalization of PLA2R1 mRNA expression in each sample type. Intron-spanning real-time PCR assays were designed with the ProbeFinder software (Roche Applied Science). All real-time PCR primers are listed in Supplementary Table 1.

### Chromatin Immunoprecipitation (ChIP) experiments

ChIP experiments were performed as described in [22] using about 30 millions of 786-0+VHL+HIF2α growing cells. HIF2α [23] or c-MYC (sc-764 X, Santa Cruz Biotechnology) specific antibodies were used for the immunoprecipitation. PCR primers for quantitative PCR are listed in Supplementary Table 2.

### Bisulphite genomic sequencing

Sodium bisulphite reactions were carried out as previously described in [24]. Two regions (nt positions −378 to +71, and nt positions +45 to +497 from the *PLA2R1* transcription start site) were analyzed. PCR amplifications were performed in 100 μl using the HotStart *Taq* DNA polymerase Kit (Qiagen) and 0.25 μM of the primers (Supplementary Table S3), after 15 min at 95°C for *Taq* polymerase activation and 35 cycles (0.5 min denaturation at 94°C, 1 min annealing at 52°C, and 1.5 min extension at 72°C). To determine accurately the proportion of methylated CpG, PCR products were cloned into a pGEM-T vector (Promega) and random clones were analyzed by automatic sequencing (Genomics, Beckman Coulter).

### Statistics

Graphs are presented with SD as error bars, and Student's t test was used to determine the p value (* p< 0.05; ** p< 0.01; *** p< 0.001) unless indicated otherwise in the figure legends.

## SUPPLEMENTARY FIGURES AND TABLES




