# Supplementary material for: Repression of PLA2R1 by c-MYC and HIF-2alpha promotes cancer growth
Source: Oncotarget. 2014 Jan 16;5(4):1004–13. doi: 10.18632/oncotarget.1681 (PMC4011578; doi:10.18632/oncotarget.1681)

Supplementary Figures Legends

Figure S1: PLA2R1 decreased favors RCC tumor growth. ACHN cells were infected with a ctrl or with shRNA targeting PLA2R1 encoding vectors, puromycin selected and injected into nude mice as described in the figure 4. (A) Photographs show a representative picture of mouse at necropsy for each condition. (B) Statistical table with at each time of measure; the number of mice, the average of tumor volume, the SEM and the p value calculated using T-test.

Figure S2: PLA2R1 expression inhibits transformation and decreases tumor growth in 786-0 cells. 786-0 cells were infected with a control or a PLA2R1 encoding retroviral vector and puromycin selected. (A) Ectopic expression of PLA2R1 was checked by Western-Blotting. Tubulin was used as loading control. (B) Twelve thousand and five hundred 786-O cells were seeded in agar and let to grow. After 1 week, colonies were counted for each indicated conditions and representative photographs are displayed. Histograms show a representative experiment from 3 independent experiments. Statistics were performed using T-test. *** represent p value< 0.005. (C) Five millions of 786-O cells were grafted in the right flank of 7 weeks old Nude mice. Tumor growth was monitored using caliper. Tumor volume was calculated using V=LxlxH/2. (D) Photographs show a representative picture of mice (upper panel) and tumor (lower panel) at necropsy for each condition. Scale bar represent 1cm. (E) Statistical table representing; the time of the measure, the number of mice, the average of the tumor volume, the SEM and the *p* value calculated using T-test.

Figure S3: PLA2R1 increased blocks ACHN tumorigenicity. ACHN were infected with a control vector or a vector encoding PLA2R1, selected and injected in nude mice as described

in the figure 6 legends. (A) Photographs show a representative picture of mice at necropsy. (B) Statistical table showing for each group; the number of mice, the tumor volume average, the SEM and the *p* value calculated using T-test.

Figure S4: PLA2R1 induces cell death and a proliferation decreased in 786-O derived tumors. Control or PLA2R1 constitutively expressing 786-O-derived tumors were collected, fixed and embedded. Tissue sections from tumors were then used to analyze proliferation and cell death for each experimental condition. (A-B) Immunohistochemistry against Ki-67 proliferation marker was performed. Nuclei were counterstained by an hematoxylin solution. (A) Histogram representing the percentage of Ki-67 positive cells was displayed. Positive cells were counted on at least 4 independent fields per tumor and at least 3 independent tumors. (B) Representative photographs are shown. (C-D) TUNEL assays were performed to label dying cells. After nuclear staining by Hoechst dye, the percentage of TUNEL positive cells was calculated (at least 3 independent tumors and at least 4 independent fields/ tumor analyzed). (C) Relative TUNEL positive cells were represented. (D) Images are shown.

Figure S1,
Vindrieux *et al*.

A

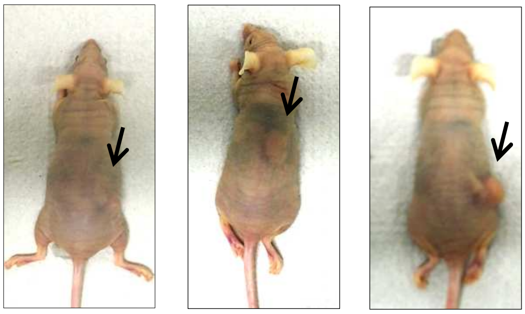

B

D16	Nb of mice	Tumor vol. (mm3)	SEM	t-Test/ctrl p value
ctrl	4	13.3	9,1	
shPLA2R1-1	5	41.9	3,5	0,02735
shPLA2R1-6	5	57.4	10,6	0,02033

D23	Nb of mice	Tumor vol. (mm3)	SEM	t-Test/ctrl p value
ctrl	4	21.2	9,1	
shPLA2R1-1	5	82.6	19.2	0.02991
shPLA2R1-6	5	117.1	32.4	0.03070

D27	Nb of mice	Tumor vol. (mm3)	SEM	t-Test/ctrl p value
ctrl	4	25.8	8.6	
shPLA2R1-1	5	111.7	23.4	0.01356
shPLA2R1-6	5	146.8	31.1	0.00878

D30	Nb of mice	Tumor vol. (mm3)	SEM	t-Test/ctrl p value
ctrl	4	46.8	14.8	
shPLA2R1-1	5	147	25.3	0.01268
shPLA2R1-6	5	196.5	31.9	0.00424

D34	Nb of mice	Tumor vol. (mm3)	SEM	t-Test/ctrl p value
ctrl	4	51.8	16.6	
shPLA2R1-1	5	172.5	35.2	0.02047
shPLA2R1-6	5	227.1	33.8	0.00230

D37	Nb of mice	Tumor vol. (mm3)	SEM	t-Test/ctrl p value
ctrl	4	62.2	20	
shPLA2R1-1	5	189.7	43.8	0.04337
shPLA2R1-6	5	254.4	31.8	0.00121

D42	Nb of mice	Tumor vol. (mm3)	SEM	t-Test/ctrl p value
ctrl	4	83.5	35	
shPLA2R1-1	5	222.8	44.2	0.05726
shPLA2R1-6	5	305.2	44.9	0.00665

D47	Nb of mice	Tumor vol. (mm3)	SEM	t-Test/ctrl p value
ctrl	4	111.8	50.3	
shPLA2R1-1	5	280.6	48.8	0.06233
shPLA2R1-6	5	352.5	57.6	0.01970

Figure S2,
Vindrieux *et al*.

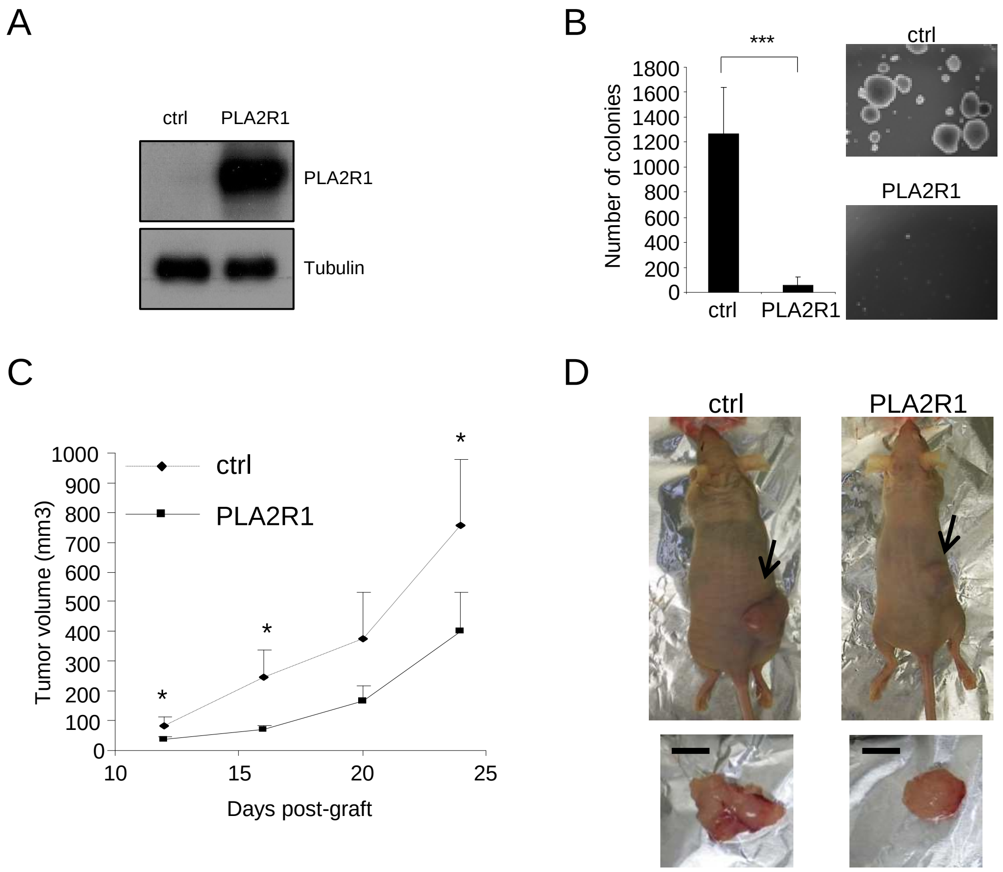

E

D12	Nb of mice	Tumor vol. (mm3)	SEM	t-Test p value
ctrl	4	45.7	14.6	
PLA2R1	4	21.1	5.1	0.019132

D16	Nb of mice	Tumor vol. (mm3)	SEM	t-Test p value
ctrl	4	117	27.3	
PLA2R1	4	36.6	5.1	0.001143

D20	Nb of mice	Tumor vol. (mm3)	SEM	t-Test p value
ctrl	4	206	101	
PLA2R1	4	94.8	31.1	0.080591

D24	Nb of mice	Tumor vol. (mm3)	SEM	t-Test p value
ctrl	4	591	248	
PLA2R1	4	257	71.4	0,041398

Figure S3,
Vindrieux *et al*.

A

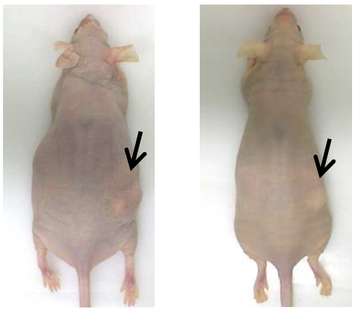

B

D19	Nb of mice	Tumor vol. (mm3)	SEM	t-Test p value
ctrl	4	75	9.5	
PLA2R1	5	34	4.5	0.00639

D25	Nb of mice	Tumor vol. (mm3)	SEM	t-Test p value
ctrl	4	105	9	
PLA2R1	5	35	7	0.00058

D39	Nb of mice	Tumor vol. (mm3)	SEM	t-Test p value
ctrl	4	132.5	18.5	
PLA2R1	5	44	5	0.00227

D60	Nb of mice	Tumor vol. (mm3)	SEM	t-Test p value
ctrl	4	217	45	
PLA2R1	5	106	13	0.04756

D75	Nb of mice	Tumor vol. (mm3)	SEM	t-Test p value
ctrl	4	265	42	
PLA2R1	5	147	28	0.05736

D89	Nb of mice	Tumor vol. (mm3)	SEM	t-Test p value
ctrl	4	351	48	
PLA2R1	5	161	45	0.0286

D104	Nb of mice	Tumor vol. (mm3)	SEM	t-Test p value
ctrl	4	391	42	
PLA2R1	5	137	25.5	0.00149

Figure S4,
Vindrieux *et al*.

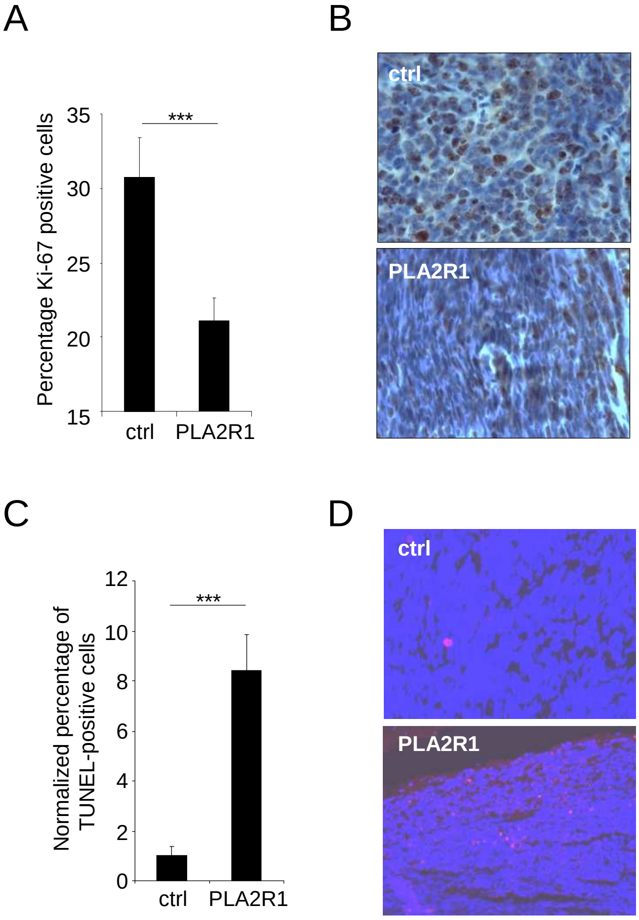

Supplement: Supplementary file 1 [file oncotarget-05-1004-s001.pdf]
