# Supplementary material for: Repression of PLA2R1 by c-MYC and HIF-2alpha promotes cancer growth
Source: Oncotarget. 2014 Jan 16;5(4):1004–13. doi: 10.18632/oncotarget.1681 (PMC4011578; doi:10.18632/oncotarget.1681)
Supplement: Supplementary file 2 [file oncotarget-05-1004-s002.pdf]

Supplementary Tables Legends

Table S1: Primers and UPL probes for performing the qRT-PCR experiments are listed.

Table S2: Primers used for the chromatin immunoprecipitation experiments.

Table S3: Primers used for PCR after sodium bisulfite modification.

Table S1,
Vindrieux *et al*.

GENE NAME		PRIMERS SEQUENCES	probe UPL Nb
PLA2R1	Forward	CATAAGTGGATTTCTTATGGGTCA	22
	Reverse	CCCTTTGATTGTATGCAAATCTT	
PPIA	Forward	CCTAAAGCATACGGGTCCTG	48
	Reverse	TTTCACTTTGCCAAACACCA	
ACTIN	Forward	ATTGGCAATGAGCGGTTC	11
	Reverse	GGATGCCACAGGACTCCAT	
VHL	Forward	CTGGAAGACCACCCAAATGT	1
	Reverse	GATGTGCAATGCGCTCCT	

Table S2,
Vindrieux *et al*.

target	sequences
PHD3 fwd	AGTGTCCGTTCCCAGCTCAG
PHD3 rev	TAGGCACAGTAAACAGGCCC
PLA2R1 PR1 fwd	TGGTCGAAGGAGAGGAAGAA
PLA2R1 PR1 rev	GTGCTGGGCCTAGACAAGAG
PLA2R1 PR2 fwd	CAGCTGGGTTCACTCTAGGC
PLA2R1 PR2 rev	GAGTTGTGGGTGAGCGATTT
PLA2R1 PR3 fwd	GTGGCTGCCTTTATTTCAGC
PLA2R1 PR3 rev	TATGGTGGGTATTGGGAGGA
PLA2R1 PR4 fwd	CATTTTCAGGCCCTGTAGGA
PLA2R1 PR4 rev	GAATCTCACATGCCCACCTT

Table S3,
Vindrieux *et al*.

positions from *PLA2R1* transcription start site		PRIMERS SEQUENCES
-378 to +71	Forward	AAAGGAGTTTAGGATTTTTTTAAAG
	Reverse	AAACCTTACCAACCCAAAACC
+45 to +497	Foward	TTTAGGGTTTTGGGTTGGTAAG
	Reverse	AAACAAAAAACTACAAAACACCACC
